# Improved therapies, incomplete data: late toxicity after modern classical Hodgkin Lymphoma radiotherapy

**DOI:** 10.2340/1651-226X.2025.44893

**Published:** 2025-11-12

**Authors:** Daniel Molin

**Affiliations:** Department of Immunology, Radiology and Pathology, research program Cancer Immunotherapy, Uppsala university, Uppsala, Sweden

**Keywords:** Lymphoma, Hodgkin disease, radiotherapy, chemotherapy, immunotherapy, mortality, morbidity

Radiotherapy, given after a limited amount of chemotherapy, remains a cornerstone in the treatment of most patients with limited-stage classical Hodgkin lymphoma (HL) and contributes significantly to the high cure rates observed. However, long-term survivors are at risk for a spectrum of late treatment-related toxicities, among which second malignancies represent a major cause of morbidity and mortality. One of the most clinically significant late effects is the risk of radiation-induced breast cancer, particularly in women who received mediastinal and/or axillary radiotherapy before the age of 30 years [1, 2]. This risk increases with time since exposure and is influenced by factors including radiation dose, volume, and the use of concurrent chemotherapy, especially alkylating agents that may modulate hormonal exposure [3].

Chamberlin et al. have thoroughly conducted a systematic review of breast radiation dose metrics reporting in lymphoma radiotherapy studies published in 2020–2023 [4]. Most included studies focused on patients with HL. Across 57 publications, they identified 34 distinct breast dose metrics, with mean breast dose (MBD) being the most commonly reported. Due to the considerable variability in how breast dose was measured and reported, pooling data across studies proved challenging. To address this, the authors propose standardized reporting guidelines to facilitate future large-scale analyses of breast radiation exposure. They emphasize the need to systematically report key factors known to influence breast dose, including whether axillary irradiation was used, given its significant impact on breast tissue exposure.

This editorial aims to provide a broader perspective on the risk of late toxicities following radiotherapy for classical HL, with a focus on the impact of contemporary, advanced radiotherapy methods. While the primary focus is on HL, many of the considerations also apply to primary mediastinal B-cell lymphoma and other mediastinal diseases treated with radiotherapy. In addition, the relationship between radiotherapy-associated toxicity and systemic therapy will be explored.

Beyond breast cancer, other late effects include cardiovascular disease, hypothyroidism, pulmonary fibrosis, and other secondary malignancies such as lung cancer [5–8]. In limited-stage HL, the radiotherapy is generally preceded by chemotherapy, often two to four cycles of doxorubicin, bleomycin, vinblastine, and dacarbazine (ABVD) [9, 10] or two cycles of escalated bleomycin, etoposide, doxorubicin, cyclophosphamide, vincristine, procarbazine, and prednisone (BEACOPPesc) followed by two cycles of ABVD [11]. Both radiotherapy and chemotherapy, such as doxorubicin (heart) and bleomycin (lungs), contribute to the diverse late side effects. Advances in radiotherapy planning, such as more accurate target definition (involved-site/involved node radiotherapy [IS/INRT[), advanced delivery techniques (volumetric modulated arc therapy [VMAT] and intensity-modulated radiotherapy [IMRT]), and proton therapy (PT), aim to reduce exposure to surrounding normal tissues and mitigate these risks. Nevertheless, survivors still require lifelong follow-up with tailored surveillance strategies, for females often including annual breast MRI and mammography starting, for example, 8 years postradiotherapy or at age 25.

Most of the information we have about late side effects comes from studies of patients treated with older radiation techniques, such as mantle field irradiation. Those large fields gave a relatively good disease control [12] but at the cost of significant late side effects. In a large study, a significantly increased risk of breast and other cancers has been reported up to 40 years after treatment. The most recent calendar period studied was 1989–2000, thereby excluding modern radiotherapy [3]. Furthermore, the risk of cardiovascular disease is also elevated up to 40 years posttreatment [6]. With the risk of malignancy and heart disease, together with other late side effects, such as pulmonary disease and muscular atrophy, these patients suffer from a significant burden of health problems. A large analysis of cause-specific mortality showed that HL patients had more than a 5-fold higher risk of death due to causes other than HL [5]. The main limitation with all this retrospective data is that the treatment given was abandoned 30 years ago. Therefore, the relevance for modern treatment decisions is limited. In addition to awareness of the side effects, it is equally important not to overestimate them, in order to make informed decisions about treatment.

The problem with achieving information on late side effects from modern radiotherapy is the lack of follow-up. For involved-field radiotherapy (IFRT), some long-term data exist, but this treatment has also been abandoned today. The modern IS/INRT techniques have too short follow-up and so do modern intensity-modulated approaches such as VMAT and IMRT. Some information can be achieved with estimating, for example, lifetime attributable risk and years of life lost, from treatment plans [13, 14]. For hard facts, longer follow-up with modern techniques is, however, needed. Until then, and when the time comes for those analyses, the conclusion of Chamberlin’s paper is important. More systematic reporting of breast and other organ dose metrics is needed.

However, some data are available on more modern radiotherapy approaches with relatively long-term follow-up. Between 1999 and 2005, patients in Sweden and Norway were treated with limited-field radiotherapy, using treatment volumes smaller than those typically used in IFRT. These patients now have a maximum follow-up exceeding 25 years, offering valuable insights into long-term outcomes in the context of more contemporary radiotherapy techniques. With a median follow-up of 16 years, no excess mortality was observed compared to the general population [15]. There was an excess morbidity concerning second cancer, cardiovascular disease, and respiratory disease. This excess morbidity was limited compared to older materials and also possibly partly explained by surveillance and chemotherapy toxicity [16]. The strength of this study lies in its ability to offer a glimpse into the potential long-term outcomes of modern radiotherapy. Its limitations include a relatively small sample size, which precludes detailed analysis of specific late effects such as breast cancer, and a follow-up period that, while substantial, remains insufficient to fully capture the incidence of very late toxicities.

Several smaller studies have been performed, in which different treatment plans were compared to estimate possible long-term risks. In a Danish study, patients with stage I-II HL treated from 2006 to 2010 (*N* = 29) were analyzed concerning mean doses to the heart, valves, and coronary arteries with INRT vs mantle fields. Mean doses were significantly lower for INRT than for mantle field treatment. The range in doses with INRT treatment was, however, substantial, and for a subgroup, they estimated a 25-year absolute excess risk of cardiac event of as much as 5.1%. This group would benefit from more advanced radiation techniques [17]. Other studies have shown a favorable lifetime attributable risk for malignancies with INRT compared to IFRT (*N* = 11) [18] and lower excess relative risk with IFRT compared to mantle therapy (*N* = 37) [19]. A large analysis from the SEER database suggests that, in patients treated with radiotherapy for limited-stage HL, treatment given prior to 2000 led to a slightly higher risk of second cancer than treatment in 2000 and later. However, the median follow-up for the whole cohort was only 7.2 years [20].

Also, there are other factors than the radiotherapy to take into account, most importantly chemotherapy. In addition to the well-known risk for heart failure [21], anthracyclines have also been reported to contribute to breast cancer risk. In a study by Neppelenbroek et al. on 1,964 female 5-year HL survivors, those treated with a cumulative doxorubicin dose of > 200 mg/m^2^ had a 1.5-fold increased risk. Breast cancer risk increased 1.18-fold per additional 100 mg/m^2^ doxorubicin [22]. This has also been shown in childhood cancer [23]. Also, a part of the cardiovascular and pulmonary excess morbidity in the Nordic material can probably be attributable to chemotherapy [16]. It is important to try to separate the effect of radiotherapy from that of chemotherapy, to be able to adjust treatment according to the results. The chemotherapy might be as important, or more important, to avoid as modern radiotherapy.

In addition to chemotherapy, immunotherapy, particularly immune checkpoint inhibitors, is now being used in the treatment of HL [24, 25]. In other lymphomas, bispecific antibodies and chimeric antigen receptor (CAR) T-cells are improving the outcome. In HL, checkpoint inhibitors are currently used in routine first-line treatment for advanced-stage disease. However, their introduction in limited-stage disease appears imminent, as clinical trials are underway [26]. These agents hold the potential to reduce the required intensity of both chemotherapy and radiotherapy while maintaining, or possibly improving, cure rates. Nevertheless, very late toxicities associated with checkpoint inhibitors remain largely unknown. This uncertainty will complicate future assessments of the relative contributions and long-term risks of the various treatment modalities used in combined therapy approaches.

When selecting primary therapy with the goal of minimizing late adverse effects, it is crucial to avoid compromising disease control. In particular, radiotherapy should not be omitted in settings where it has been shown to improve progression-free survival. Relapse often necessitates more intensive second- and subsequent-line treatments, which substantially increase the burden of long-term morbidity [8]. In the Nordic cohort, excess mortality was observed among patients who experienced relapse, unlike in all other studied subgroups with limited-stage disease treated with relatively modern radiotherapy [15].

The studies included in the analysis by Chamberlin utilized different radiotherapy techniques. Mantle fields, IFRT, ISRT, and INRT were all represented, with an increasing proportion of ISRT and INRT over time. Different radiotherapy delivery techniques, such as VMAT, IMRT, and butterfly-VMAT (BVMAT), were also used. A limited proportion (4.3%) were treated with PT. One potential risk with VMAT or IMRT is the large volumes exposed to low-dose radiation, potentially contributing to second cancer risk. PT might have an advantage in this aspect. In the present study by Chamberlin et al., the analysis suggests that more modern radiotherapy techniques may reduce MBD, such as BVMAT and PT. MBD for protons was significantly lower than for VMAT (*p* < 0.01) but not compared to BVMAT (*p* = 0.28).

Previous reports have shown that the use of deep inspiration breath hold (DIBH), PT, and the combination significantly reduced the expected years of life lost, compared to IMRT in free breathing. The lowest years of life lost were found for PT in DIBH [27]. In a comparison between pencil beam scanning PT and advanced photon therapy, PT resulted in conformal target coverage and a high rate of local control while also providing reduced dose to organs at risk for HL patients, compared with photon therapy [28]. In treated patients, the efficacy of PT seems comparable to photon therapy. PT also appears safe with the limited follow-up presently available [29, 30]. In Sweden, there is experience of PT both in clinical routine and in an ongoing phase II trial, PRO-Hodgkin [31, 32]. The trial is not yet fully recruited, but work is ongoing and has been presented at meetings, with treatment plan comparisons and estimations of lifetime attributable risks. While PFS and safety data will be available in a couple of years, long-term toxicity data will take many years to achieve.

In conclusion, the findings of Chamberlin’s paper highlight the need for more systematic reporting of dose to breast tissue. It is critical to analyze data from relevant clinical trials and real-world cohorts treated with modern radiotherapy as soon as sufficient follow-up is available. Meanwhile, methods such as estimating lifetime attributable risk should be employed to anticipate these effects before definitive clinical data emerge. For both purposes, detailed and consistent reporting of dose metrics for breasts and other organs at risk is vital. The insights gained are also relevant for other diseases, like breast cancer, lung cancer, and primary mediastinal B-cell lymphomas. Beyond understanding radiotherapy-induced late effects, we must also investigate how these effects interact with chemotherapy-related toxicities to inform balanced, evidence-based treatment recommendations.

Ongoing optimization of treatment strategies remains essential. One promising approach involves the integration of novel agents, particularly immune checkpoint inhibitors. These novel therapies will also have to be taken into account in analyses of long-term effects when introduced in primary therapy. Concurrently, continued advancement in radiotherapy techniques, such as PT and other emerging modalities, is crucial to further minimize treatment-related toxicity. It is imperative that efforts to reduce late adverse effects, for example, by omitting radiotherapy, do not compromise disease control, as relapse treatments are often associated with a substantially increased burden of long-term toxicity.

## Data Availability

NA.
